# Influence of recent cannabis use on altered spectral entropy modulation and connectivity strength in patients with psychosis

**DOI:** 10.1007/s00406-025-02004-0

**Published:** 2025-06-23

**Authors:** Vicente Molina, Álvaro Díez, Inés Fernández-Linsenbarth, Emma Osorio-Iriarte, Rosa Beño-Ruiz de la Sierra, Oscar Martín-Santiago, Claudia Rodríguez-Valbuena, Juan Carlos Fiorini-Talavera, Antonio Arjona

**Affiliations:** 1 Psychiatry Service, Hospital Clinico Universitario, Valladolid, Spain; 2 Psychiatry Department, School of Medicine, University of Valladolid, Av. Ramón y Cajal, 7, 47005 Valladolid, Spain

**Keywords:** Cannabis, Psychosis, EEG, P300, Spectral entropy, Connectivity strength

## Abstract

Cannabis use is highly prevalent in individuals with psychosis, raising concerns about its influence on brain function. Electroencephalography (EEG) studies have identified alterations in brain activity in psychosis, including changes in spectral entropy (SE) modulation and connectivity strength (CS). However, the degree to which cannabis use contributes to these alterations remains unclear. This study investigated the effects of recent cannabis use on specific EEG measures previously found to be altered in psychosis: (i) SE modulation, (ii) pre-stimulus theta and broadband CS, and (iii) baseline CS in the gamma band. We focused specifically on the immediate effects of recent cannabis use, without considering factors like tetrahydrocannabinol content, frequency of use, or age of onset. We included 93 patients with psychosis (32 recent cannabis users, 61 non-users) and 86 age- and sex-matched healthy controls (HC; all non-users). Recent cannabis use was defined as any consumption within the past week, assessed through a clinical interview and confirmed by urinalysis. Patients had diagnosis of schizophrenia or bipolar disorder. EEG data were recorded during a P300 task, and SE modulation and baseline CS were calculated. Both patient groups (cannabis users and non-users) exhibited significantly impaired SE modulation and elevated gamma and broadband CS, compared to HC. Crucially, no significant differences were found between the two patient groups in any of the EEG measures. Recent cannabis use does not appear to be the primary driver of the observed electrophysiological alterations in psychosis. Impaired SE modulation and increased CS are likely core features of psychosis itself, independent of recent cannabis exposure. This suggests that these EEG abnormalities may represent underlying vulnerability markers for psychosis. However, further research is needed to explore the potential long-term and early-onset effects of cannabis use on brain function in individuals with psychosis.

## Introduction

Cannabis is a psychoactive drug with high prevalence of use among individuals with psychosis [16]. Its primary mechanism of action involves binding to cannabinoid receptors but indirectly influences various neurotransmitter systems responsible for modulating brain activity [8, 14, 23]. Consequently, cannabis use may contribute to functional brain alterations reported in psychotic syndromes, such as the electrophysiological abnormalities found in schizophrenia and bipolar disorder. These alterations, detectable through EEG and MEG due to their high temporal resolution, are relevant for understanding the underlying neural mechanisms of these syndromes, particularly the rapidly changing dynamics of neural activity modulation associated with mental functions [3].

In this context, our research group has consistently identified a significant deficit in EEG modulation during a P300 task in individuals with psychosis. This deficit, measured using spectral entropy (SE), has been replicated across multiple studies and samples of patients [1, 19, 20]. Additionally, we observed increased pre-stimulus connectivity strength (CS) in the theta and global bands during the same task, suggesting a hyperactive baseline state [2], which was inversely correlated with SE modulation [9]. These findings suggest that a hyperactive baseline state coupled with reduced capacity for neural modulation may play a role in psychoses [5, 15], coherent with excitatory/inhibitory imbalance reported in these conditions [10, 21].

While the influence of cannabis use on these functional alterations remains largely unexplored, evidence suggests a potential link. Cannabinoids significantly affect glutamate transmission, probably affecting (at least) gamma and theta oscillations [24]. Studies have shown that recent cannabis use is associated with increased theta power and decreased beta and gamma power during resting state [28]. This is further supported by research indicating decreased gamma spectral power in healthy cannabis users compared to non-users during an auditory steady-state response (ASSRs) paradigm, without affecting N100 amplitude or inter-trial coherence [25].

This study investigated the possible short-term effects of recent cannabis use on electrophysiological measures previously identified as altered in psychosis. Specifically, and based on previous reports, we examined the impact of cannabis use in the psychotic population on the following measures: (i) SE modulation [1, 19, 20]; (ii) theta and broadband baseline CS [2]; and (iii) gamma baseline CS, given the stablished influence of cannabis on gamma oscillations [25, 28] and their potential role in schizophrenia [6, 10, 17, 26]. To achieve this, we compared these measures between individuals with psychosis who currently use cannabis, those with psychosis who do not, and healthy (non-user) controls. This initial investigation focused solely on the immediate effects of recent cannabis use in currently consumer patients, not addressing at this moment other potentially important issues such as tetrahydrocannabinol content, frequency of use and age of onset, which necessitate specific study design.

## Subjects and methods

### Study sample

The study included 93 patients with psychosis (32 current cannabis users, 61 non-users; see Table 1) and 86 age- and sex-matched healthy controls (HC; all non-users). Recent cannabis use was defined as any consumption within the past week, assessed through a clinical interview and confirmed by urinalysis. The patient group comprised 75 individuals with schizophrenia (34 first episodes) and 18 individuals with bipolar disorder, all with normal hearing. Diagnoses were established by an expert psychiatrist (VM or OM) involved in the patients’ clinical care, according to the Diagnostic and Statistical Manual of Mental Disorders, 5 th edition (DSM-5). Among the 61 recent cannabis non-users, 38 had used this drug to a variable amount during recent years, although data on frequency of consumption, age of onset, tetra-hydro-cannabinol concentration and duration of use were not available.

**Table 1:** Participant sociodemographic, clinical and electrophysiological characteristics, and group comparisons

	Patients (n = 93)		HC (n = 86)
	CNN(+) (n = 32)	CNN(-) (n = 61)	
Age (years)	28.34 (7.02)	26.30 (5.05)	26.23 (5.72)
Sex (Male: Female)	25:7	37:24	47:39
Education (years)	12.11 (3.27)**	14.27 (3.79)**	16.13 (2.35)
Parental Education (years)	10.38 (3.92)**	12.16 (3.98)*	14.14 (3.91)
Diagnosis (SZ:FE:BP)	29:10:3	46:24:15	n.a
CPZ equivalents (mg)	321.90 (192.98)	355.77 (238.61)	n.a
Illness duration (months)	40.84 (53.71)	56.61 (77.64)	n.a
PANSS–Positive symptoms	13.55 (4.68)	11.70 (4.79)	n.a
BNSS–Total	32.48 (17.56)	20.89 (18.74)	n.a
WAIS-Total IQ	86.39 (11.68)**	87.57 (14.89)**	113.55 (12.28)
BACS-Verbal learning	40,39 (7.71)**	40.30 (10.61)**	53.44 (8.83)
BACS-Working memory	17.65 (3.55)**	16.79 (4.69)**	22.39 (3.14)
BACS-Motor speed	62.65 (18.14)**	61.59 (16.22)**	73.67 (17.55)
BACS-Verbal fluency	20.35 (4.85)**	19.60 (5.07)**	27.51 (4.94)
BACS-Performance speed	45.13 (9.47)**	48.30 (13.23)**	69.96 (11.65)
BACS-Problem solving	17.23 (3.27)*	16.29 (3.58)**	18.30 (2.30)
WCST-Perseverative errors (%)	18.52 (12.21)**	14.85 (13.18)**	8.58 (3.71)
SE modulation	0.062 (0.649)**	0.185 (0.679)**	−0.492 (1.216)
Pre-stimulus CS – Theta band	0.372 (0.052)*	0.366 (0.054)	0.355 (0.031)
Pre-stimulus CS – Gamma band	0.286 (0.053)*	0.280 (0.065)#	0.264 (0.040)
Pre-stimulus CS – Broadband	0.324 (0.042)*	0.317 (0.056)	0.305 (0.034)

In the patient group columns, symbols indicate significant differences compared to healthy controls (HC) using independent samples t-tests: **p <.005, * p <.05. The # symbol indicates a trend towards significance at p =.07. Additionally, the two patient groups differed significantly in years of education (p =.013) and BNSS negative symptomatology scores (p =.016)

Exclusion criteria were: (1) neurological disease; (2) history of head trauma with loss of consciousness; (3) current substance abuse (excluding nicotine and caffeine); (4) Intelligence Quotient (IQ) below 70; (5) any psychiatric treatment (for controls); and (6) current psychiatric diagnosis other than schizophrenia or bipolar disorder (for patients). Sociodemographic, behavioral, cognitive, and clinical data are presented in Table 1. All participants provided written informed consent after receiving comprehensive study information. This study was approved by the ethical committees of all participating hospitals.

Clinical and cognitive assessments were conducted as previously described [1, 2, 19, 20]. The Positive and Negative Syndrome Scale (PANSS) [11] and the Brief Negative Symptom Scale (BNSS) [13] were used to assess positive and negative symptoms, respectively. Cognitive function was evaluated using the Brief Assessment in Cognition in Schizophrenia Scale (BACS) [12] and the Wisconsin Card Sorting Test (WCST), specifically the percentage of perseverative errors.

### EEG data acquisition and measures

Electrophysiological procedures for measuring SE and CS have been detailed in previous reports [1, 2, 19, 20].

Briefly, SE quantifies the degree of uncertainty or randomness in the EEG signal during task performance [7]. High SE values indicate a more uniform distribution of spectral content (a highly random signal), while low SE values reflect concentrated power within a narrower frequency range (a more regular signal). Thus, SE serves as a global index of EEG activity, capturing changes from the pre-stimulus to the active task windows (*i.e.,* SE modulation measure). Differences in EEG modulation between groups during the task can be assessed by comparing SE values between the windows immediately prior and posterior to a target stimulus. In this study, SE was calculated for each sensor during a P300 task (pre-stimulus and response windows), and data were summarized using principal components analysis, yielding a single factor that captured most of the variance. In HC, SE modulation values are typically negative, indicating a more regular –less entropic– signal during the response compared to the pre-stimulus window.

Connectivity strength (CS) quantifies global functional connectivity within a brain network, with higher values indicating greater synchrony. This parameter, derived from the concept of density in binary networks and represents the average edge values of all node’s connections. These connections are typically quantified using phase-locking values (PLV), which, in the context of EEG, reflect the degree of synchronization between sensors. Higher synchronization is associated with greater PLV. Thus, CS provides a single, quantitative index summarizing the overall synchrony among neural assemblies within the network. For this study, CS values were calculated during the P300 task, focusing specially on the pre-stimulus window, as this previously demonstrated significantly higher CS in patients with psychosis [2].

### Statistical analysis

Between-group differences in sociodemographic, clinical and cognitive measures were assessed by t-test or chi-square as appropriate. The effects of current cannabis use on the mentioned EEG parameters (SE modulation and baseline CS in the theta, gamma and global bands), were analyzed using a one-way analysis of variance (ANOVA). This analysis compared three groups: patients with psychosis who currently use cannabis, patients with psychosis who do not use cannabis, and HC. Post-hoc pairwise comparisons (t-test for independent samples) were conducted to identify specific group differences (or trends) where appropriate.

## Results

There were no significant differences in age or sex between the three groups (cannabis-using patients, non-using patients and HC). Similarly, no significant differences were found between the two patient groups in terms of cognitive scores, family education level, or chlorpromazine (CPZ) equivalent doses. However, both patient groups had significantly lower cognitive scores and education levels compared to HC. Cannabis-using patients had lower education level (t(91) = 1.619, p = 0.013) and higher scores on the BNSS (t(91) = 2.479, p = 0.016) than non-using patients (Table 1).

The one-way ANOVA revealed a significant main effect of group for SE modulation (F(2, 176) = 9.704, p < 0.001), with a trend toward significance for baseline CS in the gamma band (F(2, 176) = 2.698, p = 0.070) and the broadband (F(2, 176) = 2.606, p = 0.077). There was no significant group effect on theta CS (F(2, 176) = 2.379, p = 0.096).

**Fig. 1:**
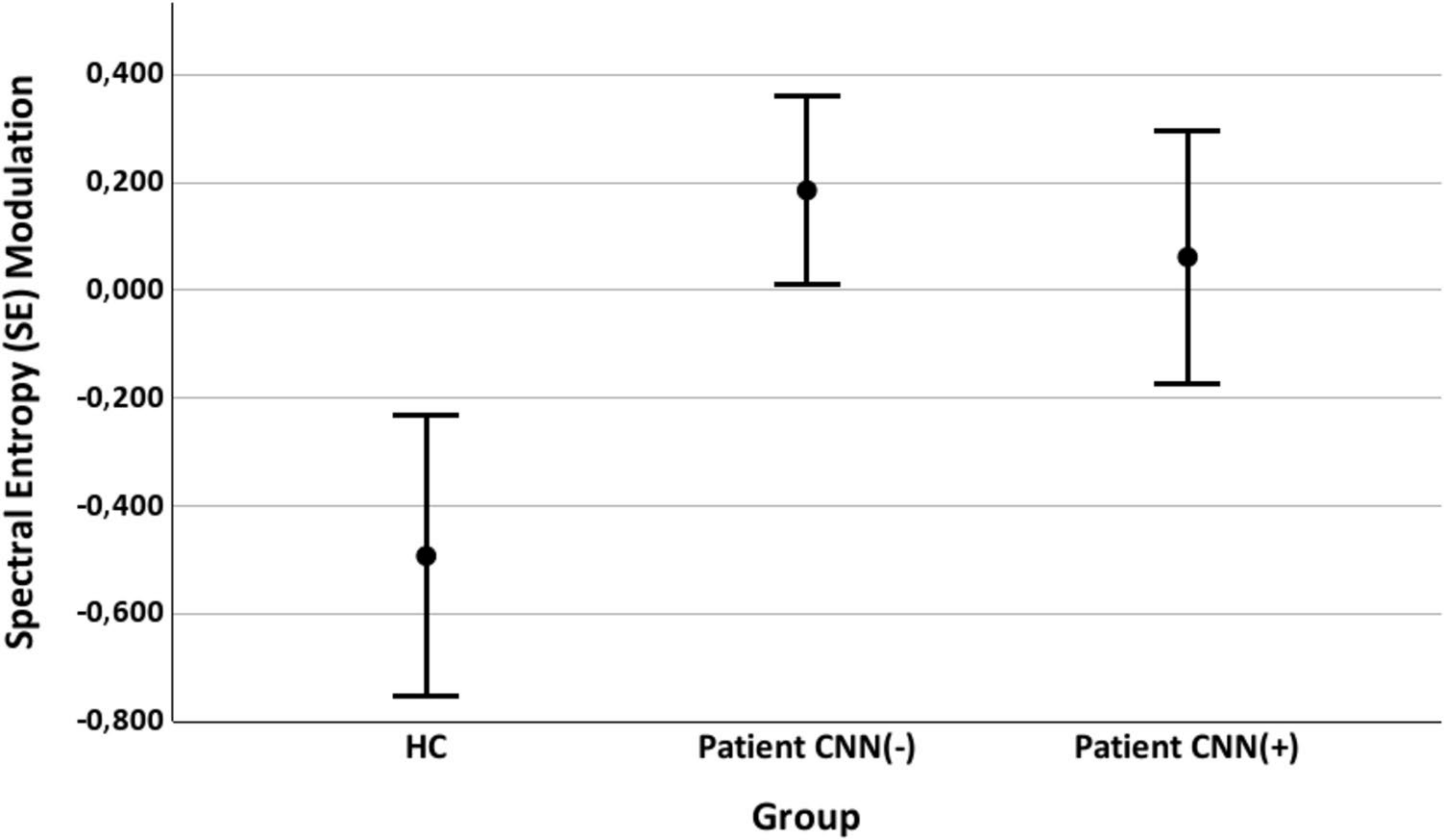
Spectral entropy (SE) Modulation values (mean and 95% confident intervals, CI) in cannabis user patients (CNN(–)), non-user patients (CNN(+)) and healthy controls (HC). Significant differences were found between each group of patients and controls, but not between both groups of patients

**Fig. 2:**
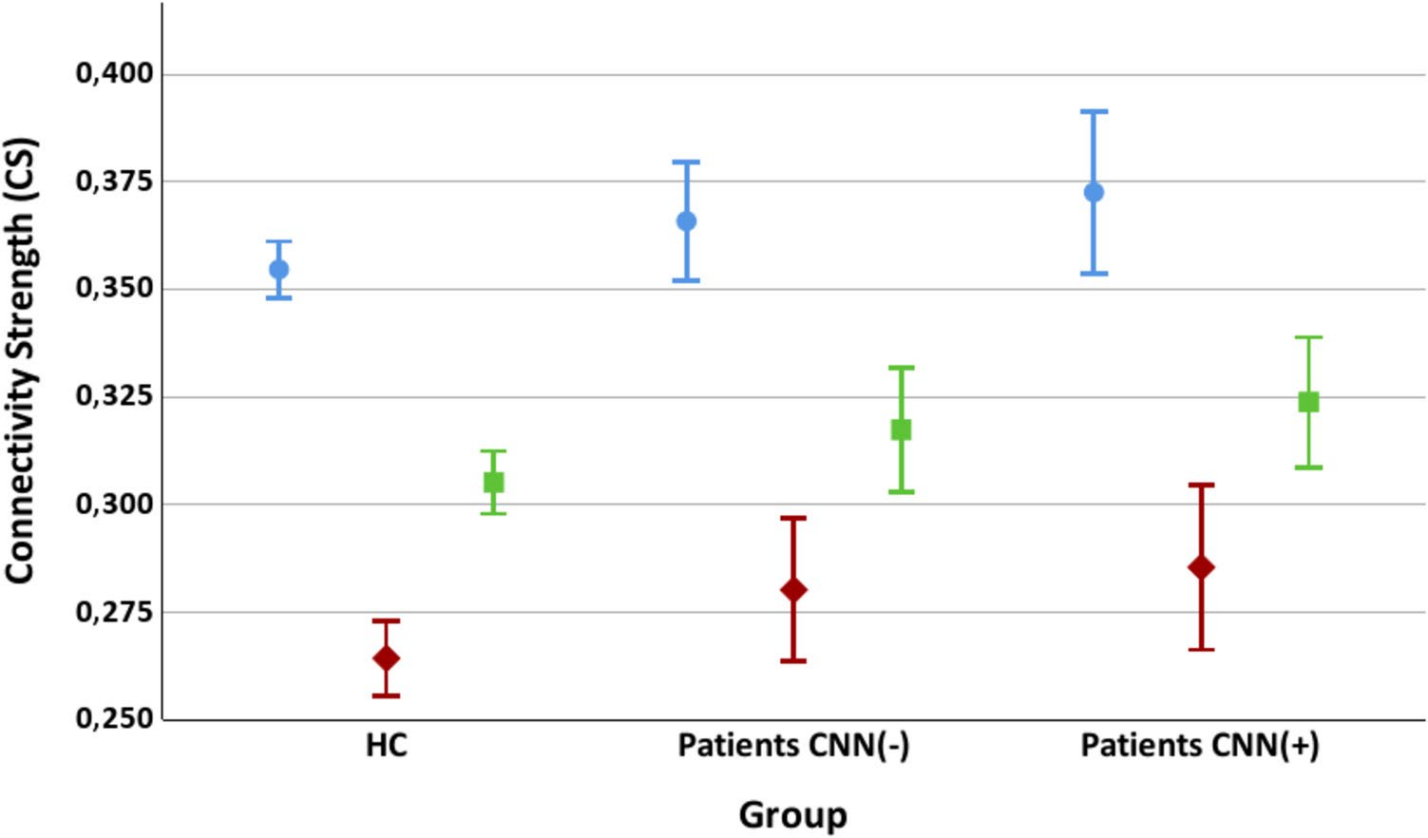
Connectivity strength (CS) values (mean and 95% confident intervals, CI) in the theta band (black), gamma band (green) and broadband (red). Significant differences or trend towards significance were found between each group of patients and controls, but not between both groups of patients

Post-hoc comparisons showed that both patient groups exhibited significantly more positive SE modulation (*i.e.,* a smaller change in SE from pre- to post-stimulus) compared to HC. This effect was observed for both cannabis users (t(116) = 3.180, p = 0.002) and non-users (t(145) = 3.937, p < 0.001) (Table 1; Fig. 1). Regarding the pre-stimulus CS parameters, post-hoc pairwise analyses found significant differences between cannabis user patients and HC for the theta band (t(116) = 2.301, p = 0.023), gamma band (t(116) = 2.320, p = 0.022) and the broadband (t(116) = 2.483, p = 0.014); and a trend between cannabis non-users and HC for the gamma band (t(145) = 1.835, p = 0.069). We found no significant differences between both groups of patients after any pairwise analysis (Table 1; Fig. 2). Thus, patients recently using or not cannabis did not differ in SE modulation or CS values.

## Discussion

Our results suggest that recent cannabis use does unlikely account for the previous findings supporting a hyperactive baseline and a deficit in task-related cortical modulation in psychosis. Specifically, we found no significant differences in SE modulation or pre-stimulus CS between patients with psychosis who used cannabis recently and those who did not. Moreover, both patient groups exhibited significantly impaired SE modulation and similarly elevated CS values compared to HC, irrespective of cannabis use. This suggests that these electrophysiological alterations are likely core features of psychosis, rather than being primarily driven by recent cannabis exposure.

Interestingly, while previous research in healthy individuals has shown that cannabis use decreases gamma activity [25, 28], our findings indicate increased gamma CS in patients with psychosis who use cannabis. This suggests that the relationship between cannabis use and gamma activity may differ in the context of psychosis, and alterations in this frequency band are unlikely explained by this substance. Furthermore, the similar pattern of elevated pre-stimulus CS in the gamma band observed in both cannabis users and non-users among our patients reinforces the idea that this alteration is likely inherent to psychosis, rather than being solely attributable to cannabis.

Our data therefore support the notion that task-related EEG modulation deficits are independent of cannabis use, at least from recent use. This aligns with our previous work, which demonstrated that these deficits are unrelated to CPZ equivalent doses and independent of treatment with antipsychotics, antidepressants, benzodiazepines and lithium, as a major effect of treatment and chronicity on these alterations [20]. Therefore, SE modulation deficit may be more closely linked or primarily related to the neurobiological underpinnings of psychosis, as suggested by its significant association with cognitive deficits [19] and baseline hyperactivity [9].

It is worth noting that some pre-existing brain structural differences, as thinner left precentral and right inferior parietal gyri and lower right caudate volume, have been reported in cannabis users before they initiate use [18]. This raises the possibility that certain brain vulnerabilities, usually related to cannabis use, may predate and potentially contribute to the onset of psychosis, rather than merely follow cannabis use. However, we cannot rule out that cannabis consumption may aggravate such brain activity alterations.

Such an (at least) relative independence between cannabis use and brain dysfunction may be relevant, since the observed pattern of baseline hyperactivity and task-related hypomodulation aligns with the concept of a relative inhibitory deficit in psychosis, which is supported by neurobiological [10] and neuroimaging research [21] in schizophrenia. Thus, while cannabis has a known effect on glutamate transmission [24], our data suggests that acute cannabis use is not a primary driver of this possible excitatory/inhibitory imbalance in psychoses.

However, while our findings suggest that acute cannabis use is not the primary factor behind SE modulation deficits or hyperactive baseline CS in psychosis, we cannot rule out the impact of long-term cannabis use or early use during adolescence, a period when cannabis use is known risk factor for schizophrenia [27]. Long-term cannabis use has been reported to influence P300 amplitude in schizophrenia patients, although both users and non-users exhibit reduced amplitude compared to HC [22].

Finally, our reliance on clinical interviews and urinalysis to assess cannabis use has limitations. These methods do not provide an objective measure of cannabis use beyond the past week, nor can they differentiate the potential influence of varying THC concentrations. Future research should address these limitations, particularly given –for example– the reported geographical variations in schizophrenia incidence linked to cannabis potency [4].

## References

[1] Bachiller A, Lubeiro A, Diez A, Suazo V, Dominguez C, Blanco JA, Ayuso M, Hornero R, Poza J, Molina V (2014) Decreased entropy modulation of EEG response to novelty and relevance in schizophrenia during a P300 task. Eur Arch Psychiatry Clin Neurosci. 10.1007/s00406-014-0525-5 25164969 10.1007/s00406-014-0525-5

[2] Cea-Canas B, Gomez-Pilar J, Nunez P, Rodriguez-Vazquez E, de Uribe N, Diez A, Perez-Escudero A, Molina V (2020) Connectivity strength of the EEG functional network in schizophrenia and bipolar disorder. Prog Neuropsychopharmacol Biol Psychiatry 98:109801 31682892 10.1016/j.pnpbp.2019.109801

[3] Dehaene S, Kerszberg M, Changeux JP (1998) A neuronal model of a global workspace in effortful cognitive tasks. Proc Natl Acad Sci U S A 95(24):14529–14534 9826734 10.1073/pnas.95.24.14529 PMC24407

[4] Di Forti M, Quattrone D, Freeman TP, Tripoli G, Gayer-Anderson C, Quigley H, Rodriguez V, Jongsma HE, Ferraro L, La Cascia C, La Barbera D, Tarricone I, Berardi D, Szoke A, Arango C, Tortelli A, Velthorst E, Bernardo M, Del-Ben CM, Menezes PR, Selten JP, Jones PB, Kirkbride JB, Rutten BP, de Haan L, Sham PC, van Os J, Lewis CM, Lynskey M, Morgan C, Murray RM, Group, E-G.W (2019) The contribution of cannabis use to variation in the incidence of psychotic disorder across Europe (EU-GEI): a multicentre case-control study. Lancet Psychiatry 6(5):427–436 10.1016/S2215-0366(19)30048-3 PMC7646282 30902669

[5] Diez A, Gomez-Pilar J, Poza J, Beno-Ruiz-de-la-Sierra R, Fernandez-Linsenbarth I, Recio-Barbero M, Nunez P, Holgado-Madera P, Molina V (2024) Functional network properties in schizophrenia and bipolar disorder assessed with high-density electroencephalography. Prog Neuropsychopharmacol Biol Psychiatry 129:110902 38036032 10.1016/j.pnpbp.2023.110902

[6] Diez A, Suazo V, Casado P, Martin-Loeches M, Molina V (2013) Spatial distribution and cognitive correlates of gamma noise power in schizophrenia. Psychol Med 43(6):1175–1186 22963867 10.1017/S0033291712002103

[7] Duff BJ, Macritchie KA, Moorhead TW, Lawrie SM, Blackwood DH (2013) Human brain imaging studies of DISC1 in schizophrenia, bipolar disorder and depression: a systematic review. Schizophr Res 147(1):1–13 23602339 10.1016/j.schres.2013.03.015

[8] Farkas I, Kallo I, Deli L, Vida B, Hrabovszky E, Fekete C, Moenter SM, Watanabe M, Liposits Z (2010) Retrograde endocannabinoid signaling reduces GABAergic synaptic transmission to gonadotropin-releasing hormone neurons. Endocrinology 151(12):5818–5829 20926585 10.1210/en.2010-0638 PMC3858799

[9] Gomez-Pilar J, de Luis-Garcia R, Lubeiro A, de Uribe N, Poza J, Nunez P, Ayuso M, Hornero R, Molina V (2018) Deficits of entropy modulation in schizophrenia are predicted by functional connectivity strength in the theta band and structural clustering. Neuroimage Clin 18:382–389 29487795 10.1016/j.nicl.2018.02.005 PMC5814380

[10] Gonzalez-Burgos G, Lewis DA (2012) NMDA receptor hypofunction, parvalbumin-positive neurons, and cortical gamma oscillations in schizophrenia. Schizophr Bull 38(5):950–957 22355184 10.1093/schbul/sbs010 PMC3446219

[11] Kay SR, Fiszbein A, Opler LA (1987) The positive and negative syndrome scale (PANSS) for schizophrenia. Schizophr Bull 13(2):261–276 3616518 10.1093/schbul/13.2.261

[12] Keefe RS, Goldberg TE, Harvey PD, Gold JM, Poe MP, Coughenour L (2004) The brief assessment of cognition in Schizophrenia: reliability, sensitivity, and comparison with a standard neurocognitive battery. Schizophr Res 68(2–3):283–297 15099610 10.1016/j.schres.2003.09.011

[13] Kirkpatrick B, Strauss GP, Nguyen L, Fischer BA, Daniel DG, Cienfuegos A, Marder SR (2011) The brief negative symptom scale: psychometric properties. Schizophr Bull 37(2):300–305 20558531 10.1093/schbul/sbq059 PMC3044634

[14] Kuepper R, Ceccarini J, Lataster J, van Os J, van Kroonenburgh M, van Gerven JM, Marcelis M, Van Laere K, Henquet C (2013) Delta-9-tetrahydrocannabinol-induced dopamine release as a function of psychosis risk: 18F-fallypride positron emission tomography study. PLoS One 8(7):e70378 23936196 10.1371/journal.pone.0070378 PMC3723813

[15] Manoach DS (2003) Prefrontal cortex dysfunction during working memory performance in schizophrenia: reconciling discrepant findings. Schizophr Res 60(2–3):285–298 12591590 10.1016/s0920-9964(02)00294-3

[16] McLoughlin BC, Pushpa-Rajah JA, Gillies D, Rathbone J, Variend H, Kalakouti E, Kyprianou K (2014) Cannabis and schizophrenia. Cochrane Database Syst Rev. 10.1002/14651858.CD004837.pub3 25314586 10.1002/14651858.CD004837.pub3 PMC10107010

[17] Metzner C, Maki-Marttunen T, Karni G, McMahon-Cole H, Steuber V (2022) The effect of alterations of schizophrenia-associated genes on gamma band oscillations. Schizophrenia (Heidelb) 8(1):46 35854005 10.1038/s41537-022-00255-7 PMC9261091

[18] Miller AP, Baranger DAA, Paul SE, Garavan H, Mackey S, Tapert SF, LeBlanc KH, Agrawal A, Bogdan R (2024) Neuroanatomical variability associated with early substance use initiation: results from the ABCD Study. medRxiv 10.1001/jamanetworkopen.2024.52027 PMC11686416 39786408

[19] Molina V, Bachiller A, Gomez-Pilar J, Lubeiro A, Hornero R, Cea-Canas B, Valcarcel C, Haidar MK, Poza J (2018) Deficit of entropy modulation of the EEG in schizophrenia associated to cognitive performance and symptoms. A replication study. Schizophr Res 195:334–342 28886890 10.1016/j.schres.2017.08.057

[20] Molina V, Lubeiro A, de Luis Garcia R, Gomez-Pilar J, Martin-Santiago O, Iglesias-Tejedor M, Holgado-Madera P, Segarra-Echeverria R, Recio-Barbero M, Nunez P, Haidar MK, Fernandez-Sevillano J, Sanz-Fuentenebro J (2020) Deficits of entropy modulation of the EEG: a biomarker for altered function in schizophrenia and bipolar disorder? J Psychiatry Neurosci 45(3):190032 10.1503/jpn.190032 PMC7850148 32100521

[21] Reddy-Thootkur M, Kraguljac NV, Lahti AC (2022) The role of glutamate and GABA in cognitive dysfunction in schizophrenia and mood disorders - a systematic review of magnetic resonance spectroscopy studies. Schizophr Res 249:74–84 32107102 10.1016/j.schres.2020.02.001 PMC7874516

[22] Rentzsch J, Stadtmann A, Montag C, Kunte H, Plockl D, Hellweg R, Gallinat J, Kronenberg G, Jockers-Scherubl MC (2016) Attentional dysfunction in abstinent long-term cannabis users with and without schizophrenia. Eur Arch Psychiatry Clin Neurosci 266(5):409–421 26182894 10.1007/s00406-015-0616-y

[23] Sherif M, Radhakrishnan R, D’Souza DC, Ranganathan M (2016) Human laboratory studies on cannabinoids and psychosis. Biol Psychiatry 79(7):526–538 26970363 10.1016/j.biopsych.2016.01.011

[24] Sherif MA, Cortes-Briones JA, Ranganathan M, Skosnik PD (2018) Cannabinoid-glutamate interactions and neural oscillations: implications for psychosis. Eur J Neurosci 48(8):2890–2902 29247465 10.1111/ejn.13800

[25] Skosnik PD, D’Souza DC, Steinmetz AB, Edwards CR, Vollmer JM, Hetrick WP, O’Donnell BF (2012) The effect of chronic cannabinoids on broadband EEG neural oscillations in humans. Neuropsychopharmacology 37(10):2184–2193 22713908 10.1038/npp.2012.65 PMC3422484

[26] Sun Y, Farzan F, Barr MS, Kirihara K, Fitzgerald PB, Light GA, Daskalakis ZJ (2011) gamma oscillations in schizophrenia: mechanisms and clinical significance. Brain Res 1413:98–114 21840506 10.1016/j.brainres.2011.06.065

[27] Tandon R, Nasrallah H, Akbarian S, Carpenter WT Jr, DeLisi LE, Gaebel W, Green MF, Gur RE, Heckers S, Kane JM, Malaspina D, Meyer-Lindenberg A, Murray R, Owen M, Smoller JW, Yassin W, Keshavan M (2024) The schizophrenia syndrome, circa 2024: What we know and how that informs its nature. Schizophr Res 264:1–28 38086109 10.1016/j.schres.2023.11.015

[28] Vahed N, Saberizafarghandi MB, Bashirpour H, Ahmadkhaniha HR, Arezoomandan R (2024) Effect of cannabis on brain activity in males: quantitative electroencephalography and its relationship with duration, dosage, and age of onset. J Clin Neurosci 132:110982 39667315 10.1016/j.jocn.2024.110982

